# Global utilization of biportal spinal endoscopy: Case series on management of lumbar pathology in Soddo, Ethiopia

**DOI:** 10.1016/j.ijscr.2024.110046

**Published:** 2024-07-20

**Authors:** William L. Sheppard, Kaleab Getachew, Temesgen Zelalem, Duane Anderson, Don Young Park

**Affiliations:** a Cleveland Clinic Foundation, Neurological Institute, Cleveland, OH, USA; b Soddo Christian Hospital, Division of Spine Surgery, Soddo, Ethiopia; c UC Irvine Department of Orthopaedic Surgery, UC Irvine School of Medicine, Orange, CA, USA

**Keywords:** Endoscopy, Lumbar vertebrae, Spine surgery, Biportal

## Abstract

**Introduction:**

Biportal spinal endoscopy is a safe and cost-effective methodology for the management of lumbar pathology in rural underserved hospitals that have standard orthopaedic arthroscopic equipment, but lack access to microscopy.

**Methods:**

This is a case series noting 1-year outcomes from 5 patients managed with biportal spinal endoscopy during an 11-day mission trip to Soddo, Ethiopia in November 2022. Surgical complications, postoperative assessments, and patient-reported outcomes (PROs) were retrospectively collected and analyzed. Surgical techniques and equipment utilized were shared with residents and faculty at the hospital.

**Results:**

Five Ethiopian patients (4:1, female:male) with no prior spine surgery history elected to undergo biportal surgery for the diagnoses of lumbar stenosis and disc herniation, averaging 31 years of age with Body-Mass-Indices (BMI) <35. Two patients underwent endoscopic discectomy, and three patients underwent endoscopic unilateral laminotomy and bilateral decompression (ULBD). There were no complications. No postoperative functional deficits, infections, readmissions, revisions, or wound dehiscences were noted at 12 months follow-up. VAS-back and VAS-leg scores improved to <2/10 for 80 % of patients. All patients returned to work/activities of daily living. No patients required postoperative pain management beyond the 2-week post-operative follow-up.

**Conclusion:**

With these five patients, we found that biportal spinal endoscopy can be safely applied in rural hospital settings with limited resources. This supports biportal spinal endoscopy as a viable minimally invasive modality for the management of lumbar pathology. For hospitals with limited resources, but access to arthroscopic equipment, biportal spinal endoscopy is a feasible option.

**Level of evidence:**

IV.

## Highlights

• Biportal spinal endoscopy may be a cost-effective option for spinal endoscopy, especially for hospitals that have arthroscopic equipment for common arthroscopic surgeries.
• Five Ethiopian patients elected to undergo biportal surgery for the diagnoses of lumbar stenosis and disc herniation.
• No postoperative functional deficits, infections, readmissions, revisions, or wound dehiscences were noted at 12 months follow-up. All patients returned to work/activities of daily living.
• The 5 biportal cases reported are the first performed in this region of Ethiopia. We report the successful use of the technique, as well as the challenges faced given the limited resources.
• For hospitals with limited resources, but access to basic arthroscopic equipment and spine surgery instruments, biportal spinal endoscopy is a feasible option.

## 1 Introduction

Biportal spinal endoscopy is increasingly utilized for the management of lumbar disc herniations and lumbar stenosis [[1], [2], [3], [4]]. Biportal spinal endoscopy is a water-based endoscopic technique that utilizes two small separate incisions, one for the endoscopic camera and the other for the surgical instruments [1,2]. The endoscopic irrigation allows for excellent visualization and the separate incisions allows for greater flexibility and versatility during the procedure [1,2]. The technique has been shown to be both efficacious and safe, with similar clinical outcomes to traditional open or microscopic techniques [1]. Recently, biportal spinal endoscopy was safely and effectively performed in both inpatient and outpatient settings [2]. More and more surgeons have learned and adopted the biportal technique across the globe, citing comparable postoperative outcomes and safety to more traditional methods [1,3,4].

Managing lumbar stenosis with the biportal technique allows central and lateral recess decompression with incisions <1 cm in length, with some authors preferring it over traditional methods due to the enhanced visualization of spinal anatomy using the endoscope [5,6]. For lumbar disc herniations, biportal has similarly been found to be as effective as traditional methods, with significant improvement of postoperative PROs [7,8]. Multiple recent retrospective studies have added to the clinical evidence demonstrating clinical effectiveness, and increasingly surgeons are learning the biportal technique to add to their surgical armamentarium [[9], [10], [11], [12]]. Park et al., showed unequivocally that biportal endoscopic decompressive laminectomy is an alternative that offers similar clinical outcomes as microscopic and open surgery [13]. Several other prospective studies and RCTs further supported these findings to give more evidence to the technique [[14], [15], [16]].

An experienced endoscopist can expect to perform 50–100 cases yearly, with the average cost savings of $8000 per QLAY (quality adjusted life year) when compared to traditional methods [17,18]. Biportal spinal endoscopy may allow hospitals provide a viable option for endoscopic spine surgery to their patient population at a significantly lower cost [18,19]. With proper training, it would take an average of 30 cases to reach technical comfort with the procedure, lowering the barrier to entry for surgeons to provide this procedure for their patients [20].

Given the cost savings, the biportal technique could be implemented for use in rural settings, locations with limited resources, or areas without direct access to an operative microscope, which can be quite costly. Many hospitals already own arthroscopic towers for common arthroscopic surgeries, reducing the need for additional equipment to perform biportal spinal endoscopy and decreasing the financial burden for hospitals with less resources [18]. In this case series, we performed biportal spinal endoscopy in a rural community hospital in Ethiopia and present the cases and their outcomes.

## 2 Methods

### 2.1 Helsinki statement

All medical research involving human subjects must be preceded by careful assessment of predictable risks and burdens to the individuals and groups involved in the research in comparison with foreseeable benefits to them and to other individuals or groups affected by the condition under investigation.

### 2.2 Ethics statement

Ethical approval provided by the governing bodies at the local hospital in Soddo, Ethiopia. The study has been approved by the Institutional Review Board (IRB). Reference number 2024.04.29.001 has been given on 4/29/2024 by the IRB in order to present our findings in this study. This decision is valid for a 2-year period. Patients have been deidentified and consented in accordance to standard practices at the local hospital. Additionally, written informed consent was obtained from the patient or legal guardian for publication and any accompanying images. This work has been reported in line with the PROCESS criteria [22].

### 2.3 Study design

This case series was retrospective in design and from a single center. We analyzed the 1-year postoperative outcomes from 5 patients managed with biportal spinal endoscopic decompression during an 11-day mission trip to Soddo, Ethiopia in November 2022. Consecutive patients underwent biportal spinal endoscopy performed by a single orthopaedic spine surgeon from the United States at the local teaching hospital. As part of an effort to introduce the technique to the region and establish an endoscopic spine program, Ethiopian orthopaedic surgery residents, orthopaedic surgery and neurosurgery attendings at the local hospital were taught how to perform biportal spinal endoscopy and served as first assist during the surgeries. Surgical complications, postoperative assessments, and patient-reported outcomes were retrospectively collected and analyzed. Surgical techniques and equipment utilized were shared with residents and faculty at the local hospital.

### 2.4 Operating room setup

**Fig. 1:**
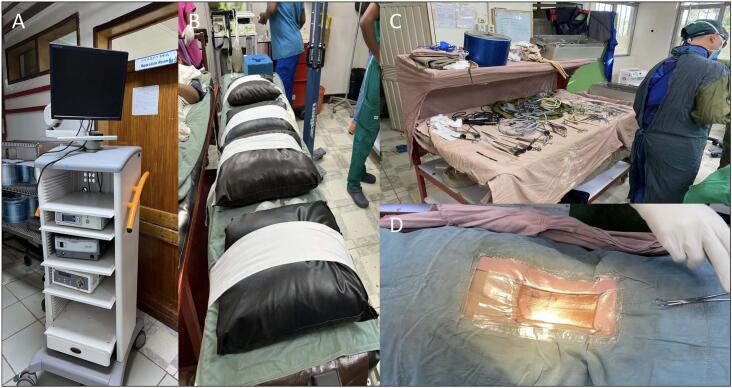
Operating room setup. (A) Standard arthroscopic tower. (B) Flat-top bed simulating the standard Jackson Prone setup utilized in the United States. (C) Standard equipment utilized during the procedure. (D) Example of pre-incisional markings made by needle localization under fluoroscopic guidance.

Each patient was assessed preoperatively with routine history and physical exam. Preoperative MRI was also obtained 1–2 weeks prior to assessment in anticipation for the United States' team arrival. Exclusion criteria included any revision surgery and any surgery for the diagnosis of spinal instability, infection, or acute trauma. After exclusion, 5 patients were selected to undergo biportal endoscopy for lumbar spine pathology. Fig. 1, shows the standard setup for biportal spinal endoscopy at Soddo. Not shown, is the standard fluoroscopic imaging system (C-arm) and the autoclave used to sterilize water used during endoscopy. No arthroscopic pump was available; thus, gravity irrigation was utilized with sterilized water. Operative technique detailed in cited studies [9].

### 2.5 Case specific presentations

All 5 patients were assessed clinically prior to proceeding with biportal spinal endoscopy by the local surgeons and the U.S. surgeon performing the surgeries at the local hospital clinic. The inclusion criteria included lumbar stenosis and lumbar disc herniations causing either neurogenic claudication or lumbar radiculopathy. Exclusion criteria included acute vertebral fractures, active osteomyelitis-discitis, uncontrolled metabolic disease and bleeding disorders, severe deformity, and instability. Patients were evaluated by the local anesthesiologist for medical clearance and optimization prior to surgery.

All advanced imaging was obtained at the local hospital through the radiology department. An intraoperative drain was placed in all cases, which is maintained until postoperative day 1. Postoperative drains were utilized to decrease the risk of epidural hematoma and is commonly used in biportal procedures [1,2,9,13]. Once removed, wounds typically are healed within 2–4 weeks. Wounds were inspected daily prior to discharge. Length of stay was typically 2–3 days in this setting. Clinical follow up was performed at the local hospital clinic by the local attending surgeon with 1-year postoperative follow-up.

### 2.6 Patient 1

**Fig. 2:**
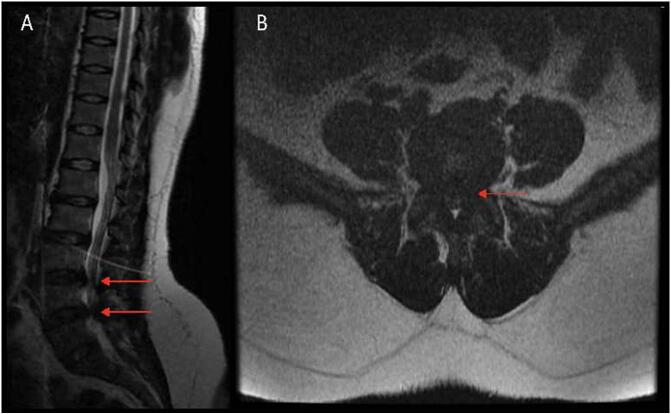
Patient 1: Preoperative MRI. (A) Sagittal T2-weighted MRI showing central stenosis at L3–4 and L4–5, with the most stenotic level being the latter. (B) Axial T2-weighted image showing significant central and lateral recess stenosis, worst at L4–5.

**Fig. 3:**
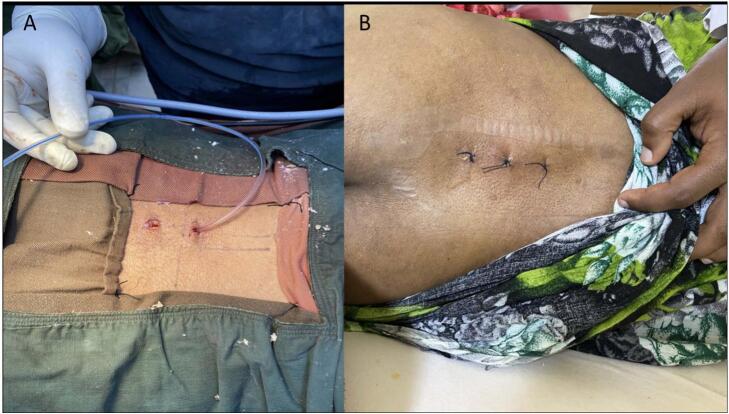
Drain placement and postoperative wound check. (A) intraoperative drain placement under direct endoscopic visualization. (B) postoperative wound check from a 2-level procedure. Incisions <1.5 cm each.

This patient is a 32-year-old female initially presenting with bilateral leg numbness, tingling, and back pain. On preoperative examination, she was noted to have full motor strength and sensation on exam without upper-motor-neuron (UMN) signs such as Hoffman, Babinski, or Clonus. Her radiating leg pain was noted in the L4 distribution. Additionally, neurogenic claudication was noted given difficulty with distanced ambulation. Fig. 2 demonstrates the preoperative Magnetic Resonance Imaging (MRI) sagittal and axial T2 weighted images. The patient has had these symptoms for well over 6 weeks, failing nonoperative management in the process. After discussing the risks, benefits, and alternatives, the patient was selected to undergo a two-level endoscopic unilateral laminotomy and bilateral decompression (ULBD) at L3–4 and L4–5. There were no complications during the procedure. Fig. 3 demonstrates drain placement, as well as the size of the incision and closure method (Single 3–0 Nylon suture, per portal).

### 2.7 Patient 2

**Fig. 4:**
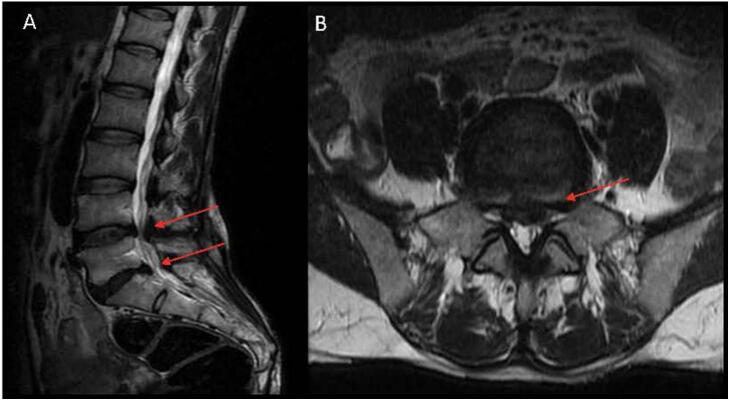
Patient 2: Preoperative MRI. (A) Sagittal T2-weighted MRI showing central stenosis at L4–5 and L5-S1, secondary to degenerative disc disease. (B) Axial T2-weighted image showing significant central/paracentral and lateral recess stenosis at L5-S1.

This patient is a 38-year-old female presenting with chronic history of neurogenic claudication after years of failed nonoperative management. Full motor strength and normal sensation were noted on exam. Positive shopping cart sign and difficulty straightening posture were also noted. She was unable to walk necessary distances required for activities of daily living. Fig. 4 is the preoperative sagittal and axial T2 weighted MRI images, showing degenerative disc disease, disc herniations, and stenosis at the L4–5 and L5-S1 levels. Similarly, to patient 1, this patient was offered a two-level ULBD. There were no intraoperative complications.

### 2.8 Patient 3

**Fig. 5:**
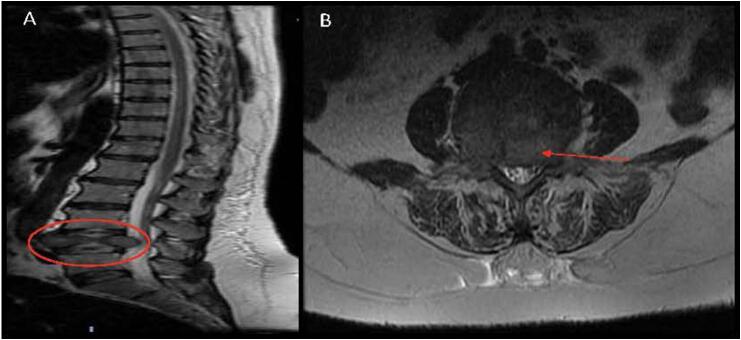
Patient 3: Preoperative MRI. (A) Sagittal T2-weighted MRI showing vertebral body collapse, retropulsion, and stenosis at the L4 level. (B) Axial T2-weighted image showing stenosis and retropulsion at the same level.

This female patient uniquely presented at 38-years-old with bilateral leg weakness and difficulty walking without assistance for approximately a month after a mechanical fall. Preoperatively, motor was 3/5 in both legs below the knee and she was wheel chair dependent. Sensation was fully intact. Fig. 5 shows the preoperative MRI, which depicts L4 vertebral body collapse with significant canal stenosis at that level. She was admitted urgently for endoscopic decompression (single level ULBD) given concerns for subacute cauda equina syndrome. There were no complications.

### 2.9 Patient 4

**Fig. 6:**
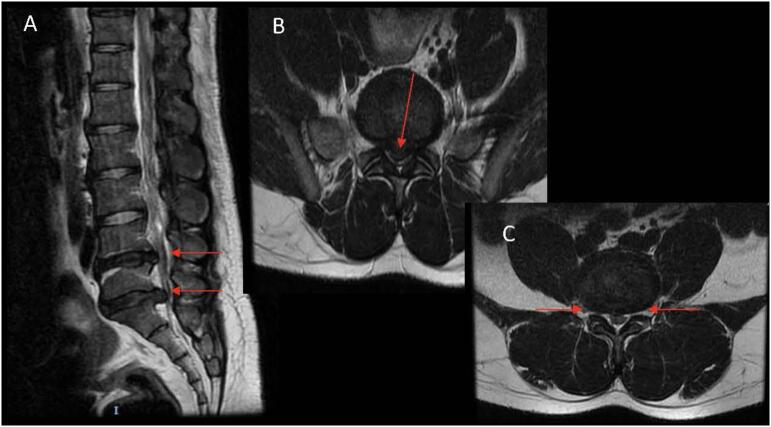
Patient 4: Preoperative MRI. (A) Sagittal T2-weighted MRI showing disc protrusion and L4–5 and disc extrusion at L5-S1. (B) Axial T2-weighted image showing stenosis at L4–5. (C) Axial T2-weighted image showing stenosis at L5-S1.

This is the only male patient of the group. This patient is a 32-year-old laborer who presented with back pain, numbness/tingling, and burning sensations in his legs. Patient had failed nonoperative management. Since onset, more the 6 weeks prior, the patient had noted improved back pain, but radicular pain in the L5 distribution persisted. Patient was indicated for a two-level biportal endodiscectomy at L4–5 and L5-S1. The most significantly stenotic level was L5-S1 due to disc extrusion as shown in Fig. 6.

### 2.10 Patient 5

**Fig. 7:**
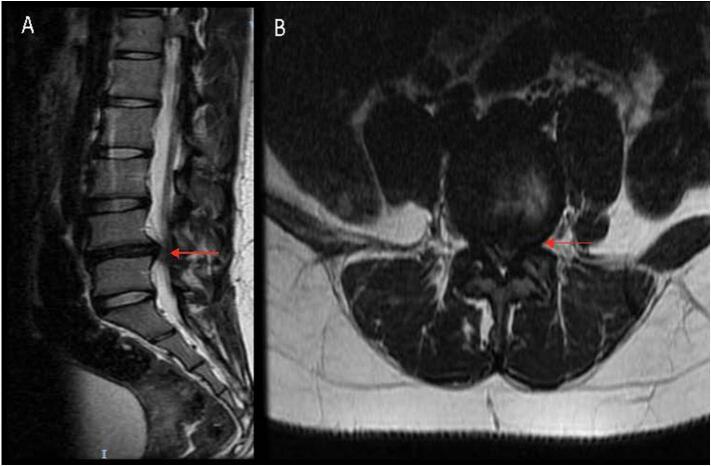
Patient 5: Preoperative MRI. (A) Sagittal T2-weighted MRI showing disc protrusion at the L4–5 level. (B) Axial T2-weighted image showing stenosis at the same level.

This 16-year-old female patient is the youngest of the group. She presented with sub-acute mild back pain and left leg radicular pain in the L5 distribution. Fig. 7 details preoperative imaging. She was given a course of nonoperative management; however, failed to improve by 6 weeks. Surgery was therefore offered [21]. After discussing the risks, benefits, and alternatives, the patient and family elected to proceed with single level biportal endoscopic discectomy. No intraoperative complications were noted.

### 2.11 Statistical methods

The primary outcomes were postoperative complications and changes in PROs at 1-year follow-up. In Soddo, standard monthly follow-up is uncommon; however, 1-year follow-up was able to be coordinated with the 5 patients residing throughout the region. Given total study population, no comparative statistical analyses were done. Changes in preoperative-to-postoperative PROs were documented. Where appropriate, analyses were performed using a 2-tailed Student's *t-*test after ensuring normal distributions. Statistical analyses were performed using Stata 16 (Stata Statistical Software: Release 16. College Station, TX: StataCorp LLC.). No demographic analyses performed, only reported, as all patients were of Ethiopian discent.

### 2.12 Preoperative/intraoperative assessments

All 5 patients are of Ethiopian descent with an average age of 31 (16–38) and a female:male ratio of 4:1. No patient underwent prior spinal surgery. All patients maintained a BMI of <35. All occupations involved manual labor, aside from the 16-year-old student. Four patients were independently ambulatory, not on chronic narcotics, and met inclusion criteria detailed above. Only one patient had significant weakness noted resulting in use of a wheel-chair for distanced travel. Patients either underwent (1) biportal endoscopic ULBD for lumbar stenosis, or (2) biportal endoscopic discectomy for acute disc herniation. Levels were determined by clinical exam and advanced imaging provided. These demographics are summarized in Table 1. Additionally, 3 biportal endoscopic ULBD and 2 endoscopic discectomies were performed for a total of 8 lumbar levels. Preoperative VAS-back and VAS-leg not formally obtained preoperatively, but noted to have 8/10 back and leg pain on average.

**Table 1:** Demographics, preoperative assessment, surgery performed.

Patient number	Age	Gender	Preoperative complaint/findings	Surgery	Levels
1	32	Female	Bilateral Leg Numbness, Neurogenic Claudication, Back Pain	ULBD	2
2	38	Female	Neurogenic Claudication, Back Pain	ULBD	2
3	38	Female	Neurogenic Claudication, Bilateral Leg weakness (3/5 motor), Chronic Compression Fracture L4 with retropulsion	ULBD	1
4	32	Male	Lumbar Radiculopathy Bilateral Lower Extremities (L5)	Biportal Endo-discectomy	2
5	16	Female	Unilateral Lumbar Radiculopathy (L5)	Biportal Endo-discectomy	1

### 2.13 Postoperative assessment

No postoperative complications were noted. No incisional infections nor revisions or return to the operating room were noted/required. Subjectively, patients were satisfied with the procedure. All patients were noted to have full strength and sensation 1-year postoperatively. All but one patient had returned to baseline levels of activity. The patient with the chronic L4 compression fracture regained full strength at the 1-year mark, and was noted to be fully ambulatory; however, a CT was obtained at an outside facility noted lytic lesions in the femur and spine, concerning for multiple myeloma. The patient is now under care of a local hematology-oncology division and is currently doing well. VAS-leg and VAS-back scores were found to be significantly improved overall.

## 3 Clinical discussion

Biportal spinal endoscopy is an emerging ultra-minimally invasive water-based endoscopic technique with successful clinical outcomes in the treatment of common lumbar pathologies in both the outpatient and inpatient setting [2]. Multiple systematic reviews, randomized trials, prospective/retrospective studies, and meta-analyses have demonstrated significant improvement in pain and disability with low complication rates with the technique [[1], [2], [3], [4], [5], [6], [7], [8], [9], [10], [11], [12], [13], [14], [15], [16], [17], [18], [19], [20]]. In Asia, and Europe, the biportal technique has been increasingly utilized for the management of lumbar pathology [[3], [4], [5], [6], [7], [8], [9], [10], [11], [12], [13], [14], [15], [16], [17], [18], [19], [20]]. Over the past few years, these techniques have gained some acceptance in the United States [1,2]. The importance of this study was to demonstrate the safety and efficacy of biportal decompression in a rural underserved area with limited resources. These are the first cases performing biportal spinal endoscopy in Ethiopia. Our goal was to teach the biportal technique to Ethiopian surgeons so that they may establish an endoscopic spine program in Ethiopia. This would help manage decompressions via minimally invasive techniques using pre-existing arthroscopic equipment, hopefully negating the need for purchasing a microscope or loupes.

This case series included 5 patients with an average age of 31-years old. Patients presented clinically in accordance to Table 1. These presenting symptoms are often multifactorial, and can be caused by several factors such as muscle sprains, trauma, tumor, accessory bones, ligamentous hypertrophy or calcification, disc herniations, osteoarthritis, scoliosis, or other forms of congenital spinal stenosis [23]. The surgeon must assess each patient thoroughly to properly indicate the patient for decompression. Once indicated, open vs endoscopic vs other minimally invasive techniques can be discussed. In this case series, three endoscopic ULBDs and two endoscopic discectomies were done. All surgeries were led by a fellowship trained orthopaedic spine surgeon with extensive experience in biportal spinal endoscopy and Ethiopian residents and attendings learning and performing the biportal technique as first-assist surgeons. All patients had satisfactory outcomes without complications. All regained full strength, with only 1 patient requiring additional workup given concern for pathologic etiologies of lumbar compression (multiple myeloma). Performing biportal spinal endoscopy in a rural setting did not increase the risk for complications, nor jeopardize outcomes as shown in Table 1, Table 2. An average VAS-leg and VAS-back reduction of 5.2 (*p* = 0.002) and 5.4 (*p* = 0.003) was noted, respectively. There were no cases of infection, epidural hematoma, drain-related complications, or wound dehiscence. Overall, all 5 patients had successful outcomes, subjectively stating they were satisfied with the procedures they received. The Ethiopian orthopaedic surgery residents and attending noted comfort with the procedure given their prior arthroscopic experience.

**Table 2:** Postoperative outcomes assessment.

Patient number	VAS-leg/back preop^a^	VAS-leg postop	VAS-back postop
1	8	4	2
2	8	2	2
3	8	5	6
4	8	1	2
5	8	2	1
	*p*-Value	0.002	0.003

a Average score noted subjectively as formal VAS-leg and VAS-back was note obtained preoperatively.

### 3.1 Limitations and unique challenges to the region

Challenges unique to our local hospital due to the lack of resources, such as the lack of arthroscopic pump irrigation, limited sterilized water, limited arthroscopic equipment such as radiofrequency wands and bone cutting shavers, and limited hemostatic agents such as Floseal and bone wax, added a greater element of difficulty. The surgical and arthroscopic disposable supplies at the local hospital are typically donated and are in short supply at the hospital. The hospital relies heavily on donations, including the arthroscopic towers and arthroscopic and surgical equipment. As with any advanced equipment, proper maintenance, upkeep, and functioning must be ensured to perform the procedure, especially given the reliance of the arthroscopic camera to visualize the spinal anatomy and the radiofrequency wands to obtain hemostasis. In addition, due to the minimally invasive nature of the procedure, a functional C-arm fluoroscopy is required for proper localization of the spinal levels. Biportal spinal endoscopy is only possible with the proper functioning of all of these essential pieces of equipment.

Sterilized water must be processed 24 h in advance and requires a significant amount of time for sterilization. There were concerns about running out of sterilized water during our cases, a precious resource that we often take for granted and required for water-based endoscopy. We used all the hemostatic agents and bone wax that was available in the hospital for the 5 surgeries, which were necessary to reduce the risk of epidural hematoma and improve the safety profile of the surgeries. Considerable advanced planning was required to ensure that we had enough supplies to perform the 5 surgeries that were planned.

Due to these limitations and the short duration of the trip, we were only able to perform these 5 biportal surgeries. More cases with more supplies and time are necessary for proper surgeon education on the biportal technique and full implementation of biportal endoscopic program. More donations of arthroscopic and surgical equipment are necessary for the hospital to perform the 30 cases necessary to overcome the learning curve [20]. To further advance biportal spinal endoscopy at our local hospital in Soddo, and Ethiopia in general, additional trips to the region are needed and required, and we are now better prepared from our valuable experience. With the lessons learned from these challenges and limitations, we believe that this case series provides valuable information on the successful utilization of biportal spinal endoscopy at a rural community hospital in Ethiopia.

## 4 Conclusion

In conclusion, we were able to successfully perform biportal spinal endoscopy for patients with lumbar stenosis and disc herniations in a rural underserved setting with limited resources. Soddo Christian Hospital served as the first location for biportal spinal endoscopy in this region of Ethiopia. All patients underwent successful procedures with good postoperative outcomes and no postoperative complications. Biportal spinal endoscopy in the rural hospital setting can be successfully and efficaciously performed. Despite some limitations, surgeons in underserved areas with access to standard orthopaedic arthroscopic equipment can routinely perform endoscopic decompressions without the financial burden of acquiring equipment microscopy. In the future, we plan to continue our partnership with our Ethiopian colleagues to build the first biportal spinal endoscopy program in the region.

## Ethical approval

The study is exempt from IRB approval given standards of practice at Soddo Hospital in Ethiopia. The name of our hospital, and all patient identifiers were removed from the manuscript entirely. An ethics statement has been added to the manuscript as requested. This included the PROCESS statement. Please contact the hospital CEO and founder Duane Anderson (duane@soddo.org) should written documentation be requested by the review committee.

## Funding

No outside funds were provided for this study.

## Author contribution

William L Sheppard: Conceptualization, Methodology, Formal analysis, Visualization, Writing-Original draft preparation, Writing- Reviewing and Editing. Kaleab Getachew: Conceptualization, Investigation, Writing- Reviewing and Editing. Temesgen Zelalem: Conceptualization, Investigation, Writing- Reviewing and Editing. Duane Anderson: Conceptualization, Investigation, Resources, Writing- Reviewing and Editing. Don Young Park: William L Sheppard: Conceptualization, Methodology, Supervision, Project administration, Writing-Original draft preparation, Writing- Reviewing and Editing.

## Guarantor

D.Y.P – who has been changed to corresponding author as requested.

## Research registration number

1. Name of the registry: Not applicable, not “First in Man”.
2. Unique identifying number or registration ID: Not applicable.
3. Hyperlink to your specific registration (must be publicly accessible and will be checked): Not applicable.

## Conflict of interest statement

The senior author is a strategic board member for Amplify Surgical, Inc., which manufactures the biportal endoscopic equipment used in this study. Other conflicts of interest for the senior author include being a consultant for Alphatec, Nuvasive, and Seaspine in the past 36 months. The remaining authors have no conflicts of interest.
